# Nanofibers and nanoparticles from the insect-capturing adhesive of the Sundew (*Drosera*) for cell attachment

**DOI:** 10.1186/1477-3155-8-20

**Published:** 2010-08-18

**Authors:** Mingjun Zhang, Scott C Lenaghan, Lijin Xia, Lixin Dong, Wei He, William R Henson, Xudong Fan

**Affiliations:** 1 Department of Mechanical, Aerospace and Biomedical Engineering, University of Tennessee, Knoxville, TN 37996, USA; 2 Department of Electrical and Computer Engineering, Michigan State University, East Lansing, MI 48824, USA; 3 Department of Materials Science and Engineering, University of Tennessee, Knoxville, TN 37996, USA; 4 Advanced Microscopy Center, Michigan State University, East Lansing, MI 48824, USA

## Abstract

**Background:**

The search for naturally occurring nanocomposites with diverse properties for tissue engineering has been a major interest for biomaterial research. In this study, we investigated a nanofiber and nanoparticle based nanocomposite secreted from an insect-capturing plant, the Sundew, for cell attachment. The adhesive nanocomposite has demonstrated high biocompatibility and is ready to be used with minimal preparation.

**Results:**

Atomic force microscopy (AFM) conducted on the adhesive from three species of Sundew found that a network of nanofibers and nanoparticles with various sizes existed independent of the coated surface. AFM and light microscopy confirmed that the pattern of nanofibers corresponded to Alcian Blue staining for polysaccharide. Transmission electron microscopy identified a low abundance of nanoparticles in different pattern form AFM observations. In addition, energy-dispersive X-ray spectroscopy revealed the presence of Ca, Mg, and Cl, common components of biological salts. Study of the material properties of the adhesive yielded high viscoelasticity from the liquid adhesive, with reduced elasticity observed in the dried adhesive. The ability of PC12 neuron-like cells to attach and grow on the network of nanofibers created from the dried adhesive demonstrated the potential of this network to be used in tissue engineering, and other biomedical applications.

**Conclusions:**

This discovery demonstrates how a naturally occurring nanofiber and nanoparticle based nanocomposite from the adhesive of Sundew can be used for tissue engineering, and opens the possibility for further examination of natural plant adhesives for biomedical applications.

## Background

**Figure 1:**
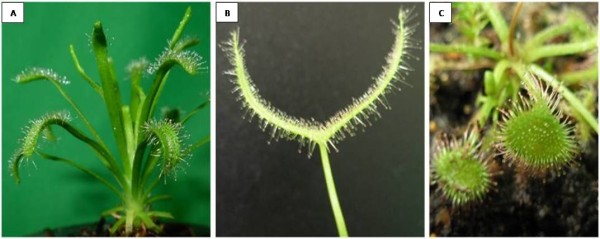
**Pictures of three species of the Sundew**. A) *D. capensis*. B) *D. binata*. C) *D. spatulata*. The leaves of each species are covered by small tentacles that generate the adhesive. This adhesive is secreted externally, allowing for easy collection.

For centuries, carnivorous plants have fascinated researchers and stimulated the minds of many scholars, including Charles Darwin. One of the carnivorous plants that interested Darwin was the Sundew (*Drosera*). The Sundew relies on complex trapping mechanisms to capture insects, which provide increased nitrogen levels that give it a competitive advantage over non-carnivorous plants [1]. Each of the Sundew tentacles secretes a small "bubble" of adhesive that fully covers its head (Figure 1). When an insect becomes stuck to the adhesive bubble, the movement of the insect generates a series of action potentials along with the tentacles, which trigger the tentacles to bend inward [2,3]. The bending brings the insect into a closer contact with other tentacles, including shorter specialized tentacles that further trigger the leaf to secrete digestive enzymes [4-9]. Digestion serves as a signal to release hormones that allow the leaf blade to curl tightly around the prey for complete digestion and absorption of nutrients [10]. This complex trapping mechanism uses the unique properties of the adhesive for capturing insects.

One of the unique properties of the Sundew adhesive is its highly elastic nature that allows it to be drawn into threads up to one meter in length [11]. Early studies confirmed that the chemical structure of the adhesive was an acid polysaccharide containing various concentrations of sugars and acids, depending on the species [11,12]. Isolation of *D. capensis *adhesive through gel filtration, cellulose acetate filtration, ion-exchange chromatography, and ultracentrifugation yielded one macromolecule with a molecular weight of 2 × 10^6 ^Daltons [11]. It was discovered that the adhesive was formed by xylose, mannose, galactose, glucuronic acid, and ester sulfate in the ratio of 1:6:6:6:1 [11]. In other species, the acid polysaccharide was found to have different ratios of chemicals. *D. binata *was reported to contain arabinose, xylose, galactose, mannose, and glucuronic acid in a ratio of 8:1:10:18:17 [12]. Further analysis also found that these polysaccharides consisted of an abundance of metal cations, including 22 mM Ca^++^, 19 mM Mg^++^, 0.9 mM K^+^, and 0.2 mM Na^+ ^in *D. capensis*. The *D. capensis *adhesive was composed of water (96%) and acid polysaccharide (4%) [11]. The ratio of polysaccharide to water has proven to be crucial in the formation of the unique elastic properties of the adhesive, as seen with other polymers [13-17]. Due to the difference in chemical composition, varying material properties were expected for different Sundew species. Environmental factors and prey availability could have imparted selection pressure that influenced the development of the adhesives over the course of evolution.

In addition to chemical composition, nanoscale morphology also contributes to the physical properties of materials. Preliminary studies on structural properties of polysaccharide-based adhesives have been conducted [18,19]. However, the relationship of the nanoscale morphology to the physical properties of adhesives remains largely unexplored. We report here our recent discovery of a nanofiber and nanoparticle-based network from the Sundew adhesive, and explore the potential of using this network for cell attachment.

## Materials and methods

### Plants

The Sundew species (*D. binata, D. capensis*, and *D. spatulata) *were purchased from the Carnivorous Plant Nursery, Derwood, MD, USA and maintained in mineral depleted soil with distilled water. The Sundew are sensitive to high concentrations of minerals, and thus it was necessary to ensure that tap water was not given to the plants. The plants were exposed to direct sunlight for 12 hour periods, and maintained at a constant temperature of 21°C. After a period of one week, all plants began to produce adhesive on the tentacle heads. It should be noted that there is no variation in the chemical composition of the adhesive from tentacle to tentacle within a species [11,12].

### Sample preparation

As shown in Figure 1, a small amount of adhesive forms on the head of each tentacle on the leaf surface. To coat a surface with this adhesive, the sample (silicon wafer, glass coverslip, and mica) was held with sterile forceps and gently brushed against the tentacle heads, allowing the adhesive to be transferred to the sample. Using this method, a different pattern of coating was achieved with each treatment. Due to the non-uniformity of the coating method, over six replicates for each species and substrate were examined. After applying the adhesive to the substrate, the samples were allowed to dry for 24 hours under a bio-safety cabinet.

Due to the large surface area of the 25 mm^2 ^coverslips, for cell attachment studies, the coverslips were cut to 5 mm^2 ^with a diamond etched pen. These smaller coverslips were then cleaned by sonication in acetone, ethanol and deionized water. Using these smaller coverslips, it was possible to more easily coat the entire surface area. To ensure that the coating covered the entire surface, an Alcian Blue pH 2.5 Periodic Acid Schiff Stain (Chromaview^®^) was applied to all coated samples per the manufacturer's instructions. With this staining procedure, acid polysaccharide stains bright blue and neutral mucosubstances stain pink. Upon completion of staining, the samples were imaged using an Olympus Fluoview 1000 microscope to visualize the stained adhesive.

In addition to the stained experimental samples, control samples were prepared for the cell attachment experiments using uncoated coverslips, and 0.1% poly-L-lysine (Electron Microscopy Sciences^®^) coated coverslips. PC12 and primary nerve cells have been shown to strongly attach to poly-L-lysine coated surfaces, but not to bare glass, so these uncoated and poly-L-lysine coated samples served as positive and negative controls. After the coverslips were coated with the adhesive, the samples were UV sterilized while submerged in Hank's Balanced Salt Solution, Formula III (Electron Microscopy Sciences^®^) for 15 minutes in a biosafety cabinet. Upon sterilization, the samples were seeded with PC12 cells in F12-K medium supplemented with 15% horse serum and 2.5% fetal bovine serum at a density of 5 × 10^4 ^cells/cm^2^. The cells were then incubated on the samples for 24 hours in a 37°C incubator with 5% CO_2 _to allow for attachment. After 24 hours, the samples were gently washed with sterile Milonig's Phosphate Buffer (Electron Microscopy Sciences^®^) warmed to 37°C. This prevented detachment due to temperature induced stress. Cells were then stained for 30 minutes with a live/dead viability dye containing calcein AM and ethidium homodimer-1 from Invitrogen (catalog number #L3224), live cells stained green and dead cells stained red. The samples were then washed and visualized using the fluorescent microscopy. Four fields of view under a 10× objective (0.0391 mm^2^) were used to determine the number of attached cells on each sample. The number of viable cells was determined by counting 100 cells at random and scoring as either alive or dead using the viability dye.

### Atomic force microscopy

AFM imaging was conducted using both an Agilent 5500 AFM and an Agilent 6000 ILM/AFM. The purpose of using both systems was to control for potential artifacts, and to allow for microscopic imaging of the samples to determine the targeted scanning areas. In addition, all samples were examined by two independent investigators who prepared their samples separately to further eliminate the possibility of artifactual data. All imaging for both systems was conducted in air in AC mode. Both systems were equipped with intermittent contact mode tips, Budget Sensors^® ^Tap150AL-G, with aluminum reflex coating. The tips had a resonant frequency of 150 kHz and a force constant of 5 N/m. Due to tip variation, manual sweeps were conducted on all tips prior to scanning to determine the actual frequency of the tip. Prior to scanning, a calibration grid was used to assure that the distance measurements of the Picoview^® ^software were accurate. Publication quality scans were conducted at a scan speed of less than 1 ln/s and a resolution of 1024 × 1024 pixels.

### Transmission electron microscopy

Transmission electron microscopy (TEM) imaging and energy-dispersive X-ray spectroscopy (EDS) were conducted using a JEOL 2200 FS TEM with attached EDS at the Advanced Microscopy Center of Michigan State University. Copper grids were coated with ultra thin carbon films. By using the thin film copper grids, the sample could be deposited on the film, instead of falling through the mesh of the grid. Grids were then coated with the Sundew adhesive in the same manner using the technique described earlier. Briefly, the copper grids were grasped using sharp electron microscopic forceps and gently brushed against the tentacles of the Sundew. After coating with the adhesive, the samples were dried overnight for subsequent analysis.

## Results and Discussion

The first stage of this study focused on determining the nanoscale structure of the dried adhesive on a variety of substrates. By determining the nanostructure of the adhesive, we could evaluate the potential uses for this material. Three Sundew species, *D. binata, D. capensis*, and *D. spatulata*, were chosen for this study. Adhesive from the tentacles from the three species were streaked onto silicon wafers, mica, and glass coverslips. After the samples were allowed to dry overnight in a biosafety cabinet, the samples were scanned using AFM.

**Figure 2:**
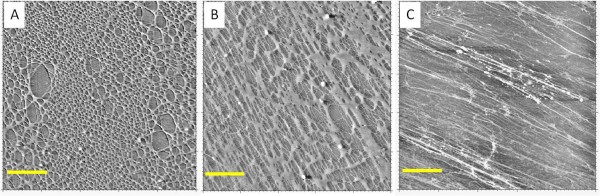
**AFM images of the Sundew adhesive for three Sundew species**. AFM scans of different species of Sundew, *D. Binata *(A), *D. capensis *(B), and *D. spatulata *(C). In each scan the network of nanofibers can be observed. Although variations can be seen in the networks from the different species, the variability in coating makes it difficult to draw significant conclusions between the species. All scans are 10 × 10 μm. Scale bar = 2 μm.

Based on the AFM analysis, it was determined that a complex network of nanofibers of varying lengths and thicknesses were deposited on the coated substrates, as shown in Figure 2. A network of nanofibers was formed from the deposition of the adhesive in all examined species (Figure 2A-C). The networks had gaps ranging from 500 nm to several microns between the nanofibers, which provided an ideal morphology for the attachment of cells. The adhesive from all species was capable of forming the observed networks on all of the tested substrates, despite their varying surface properties. From this evidence, it was determined that a complex network of nanofibers was created by streaking the adhesive from all tested Sundew species onto a variety of surfaces.

**Figure 3:**
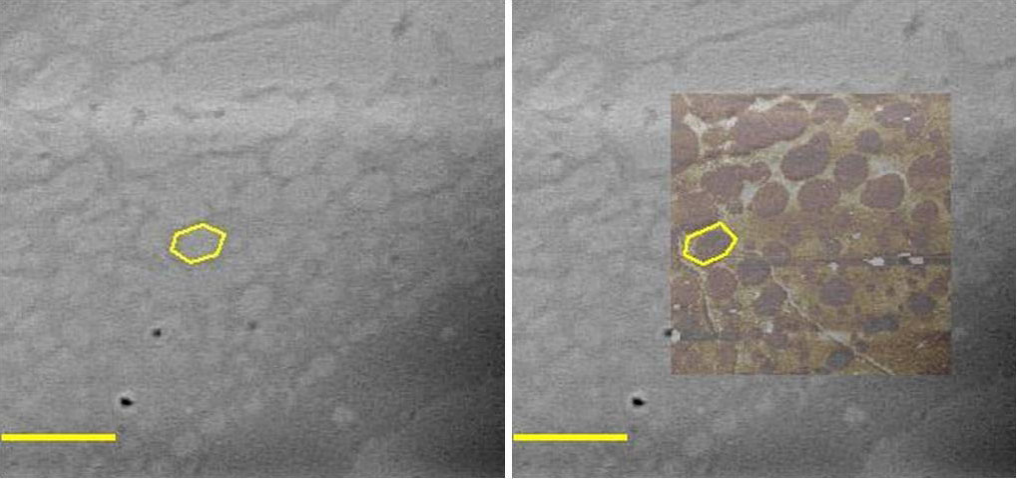
**AFM overlay of Alcian Blue stained Sundew adhesive**. Left, an Alcian blue stained sample showing the pattern of the deposited Sundew adhesive. Right, an AFM scan overlaid onto the stained micrograph. An area of interest has been outlined to demonstrate the overlap between the Alcian blue stain and the topography image from the AFM. Scale bar = 20 μm.

In order to determine if the network observed by AFM was, in fact, due to the polysaccharide component of the adhesive, a staining procedure was used to correlate the stained polysaccharide to the imaged network of nanofibers. The surface of tentacle streaked coverslips was stained with Alcian blue, pH 2.5, and Schiff reagent. This staining procedure stains acid polysaccharides blue, and neutral polysaccharides pink [20]. Using the Picoview^® ^software package, the images obtained from a large scan of the network structure was over-laid onto a light micrograph. Using this technique, it was confirmed that the network of nanofibers from the AFM scans matched the pattern of staining for the acid polysaccharide (Figure 3). From this experiment, it was clear that the networks observed in the AFM scans were the dried polysaccharide from the streaked tentacles. Using this technique, it was not possible to compare individual nanofibers, since these fibers cannot be imaged by light microscopy. However, bundled fibers were clearly correlated with the polysaccharide stain. In addition to nanofibers, nanoparticles were also observed from the AFM images.

**Figure 4:**
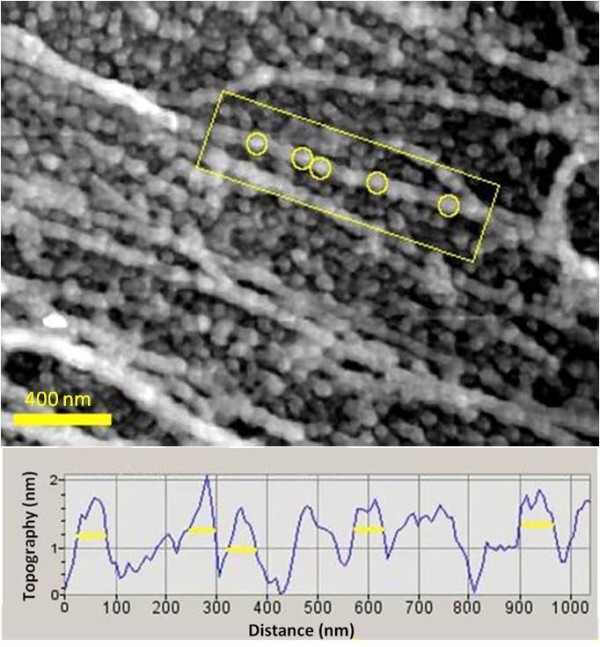
**Nanoparticle Size Characterization**. Top, an AFM image of the Sundew adhesive. Individual nanoparticles corresponding to peaks observed in the vertical cross-section are identified by yellow circles. Bottom, a vertical cross-section through the nanofiber outlined by the yellow box at the top image. Diameters of the nanoparticles were calculated based on the diameter of the observed peaks using the Picoview^® ^software package. Broader peaks indicate a group of nanoparticles that could not be individually resolved with AFM. All nanoparticles were in the range of 50-70 nm.

Smaller scan regions revealed that the nanofibers were composed of individual nanoparticles as shown in Figure 4. Nanoparticles were found in close contact with one another and were associated with the polysaccharide nanofibers. Vertical cross-sections through individual nanofibers confirmed that the nanoparticles were of a uniform size and shape with diameters in the range of 50-70 nm. In other natural systems, such as ivy, mussels, and barnacles, nanoparticles have proven to be an important component of adhesives [21,22]. It is believed that these nanoparticles are a crucial component to the generation of the material properties observed in these adhesives. The discovery of nanoparticles within the Sundew adhesive provides another example of the conserved approach used by natural systems to create nanocomposite adhesives.

**Figure 5:**
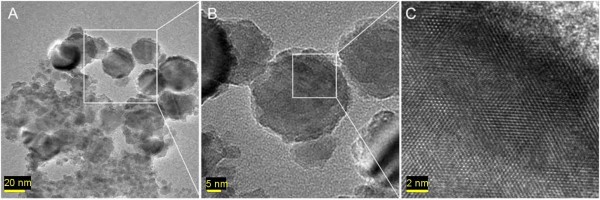
**TEM images showing the crystalline structure of the nanoparticles**. A) Agglomerations of nanoparticles with typical diameters around 35 nm (25 to 44 nm). B) The particle in the center of the image was 38 nm in diameter. C) Higher magnification demonstrating the crystalline structure of the previous nanoparticle.

In order to determine if the nanoparticles were metallic, the adhesive from each of the Sundew species was further analyzed using high resolution transmission electron microscopy (HRTEM) (JEOL 2200FS, 200 kV). If the nanoparticles were metallic, then when imaged by HRTEM, chains of nanoparticles would be observed that would correlate with the observed fibers seen by AFM. By using HRTEM, the polysaccharide would not be visualized, along with any organic nanoparticles because they are not electron dense and would be broken down by the high energy beam. After imaging of multiple samples it could be concluded that the nanoparticles observed from the AFM imaging experiments were organic and not metallic. There were no chains of nanoparticles similar to what was observed in the AFM scans. Diffuse crystalline nanoparticles were observed in several of the samples, but these nanoparticles were in a low abundance and tended to agglomerate (Figure 5). Figure 5A-C shows HRTEM images of solid nanoparticles, where the quasi-single crystalline structures of the nanoparticles can be clearly identified. Figure 5A shows several nanoparticles in the range of 25-44 nm from *D. spatulata*. The size range of these particles was below that observed for the nanoparticles imaged by AFM, 50-70 nm. By examining a single nanoparticle at high magnification, it was possible to observe the crystalline structure of the nanoparticle (Figure 5C). Since no staining was conducted on the specimen, the crystal structures observed from the nanoparticles were believed to be from the secreted adhesive. Each sample was prepared and analyzed in triplicate to rule out the possibility of environmental contamination. After identifying these diffuse crystalline nanoparticles on the grids, the next step is to determine the chemical components of these nanoparticles, in order to determine their function in the adhesive.

**Figure 6:**
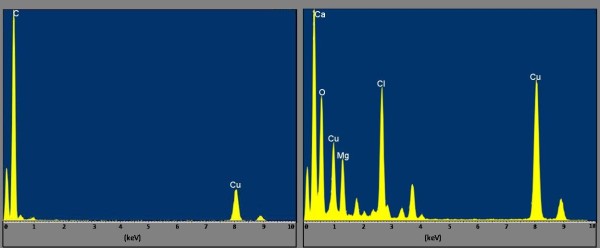
**EDS spectra of control and nanoparticle samples **Left, EDS spectrum of a control region with no nanoparticles. Chemical components include C, Cu, and small amounts of O and Si. Both Cu and C are from the grid and grid coating respectively. Right, EDS spectrum of a nanoparticle. Chemical components include Ca, Mg, O, and Cl. The presence of high amounts of Ca, Mg, and Cl, along with the crystalline structure of the nanoparticles indicates that the nanoparticles are the results of salts in the adhesive.

To achieve this goal we used EDS, a technique to determine the chemical component of samples in electron microscopy [23-25]. Analysis revealed mainly Ca and Cl in relatively high abundance from the solid crystalline nanoparticles. The solid nanoparticles were likely the result of calcium chloride, a common salt, excreted by the Sundew into the adhesive. For comparison, an EDS spectrum of a region that had no nanoparticles present was obtained as shown in Figure 6. Chemical components of this region included C, Cu, and a small amount of O and Si, where Cu is from the grid, C is mainly from the carbon film on the grid, while O and Si are from the dried solution. EDS of the crystalline nanoparticles revealed mainly Ca, Mg, and Cl, which could be indicative of biological salts present in the adhesive (Figure 6). From earlier studies focused on isolation of the Sundew polysaccharides, it was known that Ca, Mg, and Cl could be isolated from the adhesive in millimolar concentrations [11]. Our findings through HRTEM analysis revealed similar results through identification of crystalline nanoparticles that correlated to Ca and Mg salts. The concentration of salts present within each adhesive is crucial to the cross-linking potential of the polysaccharide, and contributes to the unique material properties.

**Figure 7:**
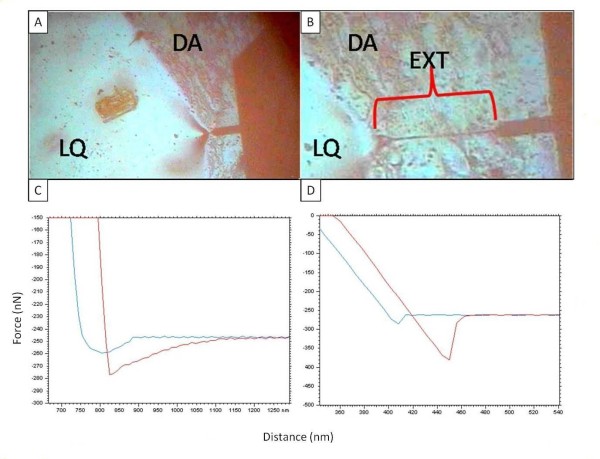
**Measurements of extension from the liquid and dried Sundew adhesive**. A) Attachment of the cantilever to the surface of the liquid adhesive. B) Horizontal extension of the liquid adhesive achieved by manually moving the cantilever in the X-direction with the stage controls. C) Force curve generated on a bare silicon wafer with an extension of 49.2 nm. In both force curves, the blue line is the approach curve, while the red line is the retraction curve. D) Force curve from the Sundew scaffold shows the extension length of 320.6 nm.

After determining the basic structural components of the adhesive, it was necessary to determine the material properties of the adhesive. The first material property tested was the elasticity of the liquid adhesive. An AFM was employed in acoustic mode (AC) with a stop at 85% to land the tip of the cantilever on the surface of the adhesive without indenting into the adhesive. Once on the surface, force spectroscopy was employed to gently indent into the liquid adhesive in nanometer increments. After indenting less than 20 nm into the adhesive, the cantilever tip was unable to withdraw from the adhesive, due to a limited vertical withdraw distance of 3 μm. As shown in Figure 7A-B, the cantilever had to be manually moved in the horizontal direction to break the cantilever-adhesive interaction. In fact, the adhesive was stretched 246 um before breaking off from the tip. Considering that the contact area between the tip and the adhesive was less than 78.5 nm^2^, the elasticity of the Sundew adhesive is quite large. Since the maximum vertical withdraw distance setting for the AFM used in this study was only 3 um, it was not possible to generate force curves from the fresh liquid adhesive due to its high elasticity. Instead, we chose to study the elastic properties of the dried adhesive applied to a surface.

To investigate whether the elastic properties were maintained from the liquid to the dried adhesive, force versus distance curves were generated on the dried adhesive. Since the adhesive was completely dried before conducting the AFM studies, there was no adhesive force observed from the network of nanofibers when compared to the bare silicon surface. However, as seen in Figures 7C-D, there was a significant increase in extension length. The extension from the dried adhesive was 320.6 nm, while the extension from the bare silicon surface was less than 49.2 nm. Similarly, the adhesive showed significant deformation compared to the bare silicon wafer. It is important to point out that the AFM experiments indicated that the dried adhesive adhered to the silicon wafer, and could not be removed using sharp probes in contact mode with fast scanning speeds (> 3 ln/s) and a negative setpoint. It is believed that a curing process takes place during drying that forms a strong bond between the adhesive and the substrate surface. This phenomenon is common for many epoxies, glues, and adhesives, where drying or chemical modification of a liquid adhesive often leads to the formation of tight bonding between the dried adhesive and the contact surface [26-30]. The stability of the dried adhesive on the surface, combined with the non-toxic components of the adhesive (salts, polysaccharide, and organic nanoparticles), and the porous network structure of the nanofibers, led to the hypothesis that the network could be used for applications in tissue engineering and wound healing.

To validate this hypothesis, it was essential to demonstrate that the Sundew network was capable of supporting cell growth. To test this ability, PC12 cells were chosen as a model system for nerve cell growth. PC12 cells were derived from a pheochromocytoma of the rat adrenal medulla [31], and are typically used as a model system for nerve cell growth and differentiation [32-34]. Three treatments were tested to determine if the network of nanofibers was capable of supporting cell attachment. Since PC12 cells do not attach to bare glass, this sample was used as a negative control. A positive poly-L-lysine coated control was used to determine the maximum number of cells that could attach on an ideal substrate. The third sample was a Sundew adhesive coated glass coverslip, stained with Alcian Blue to visualize the pattern of staining. Viability was determined by using a calcein/ethidium bromide live/dead assay and all samples were imaged using an Olympus Fluoview 1000 confocal microscope.

**Figure 8:**
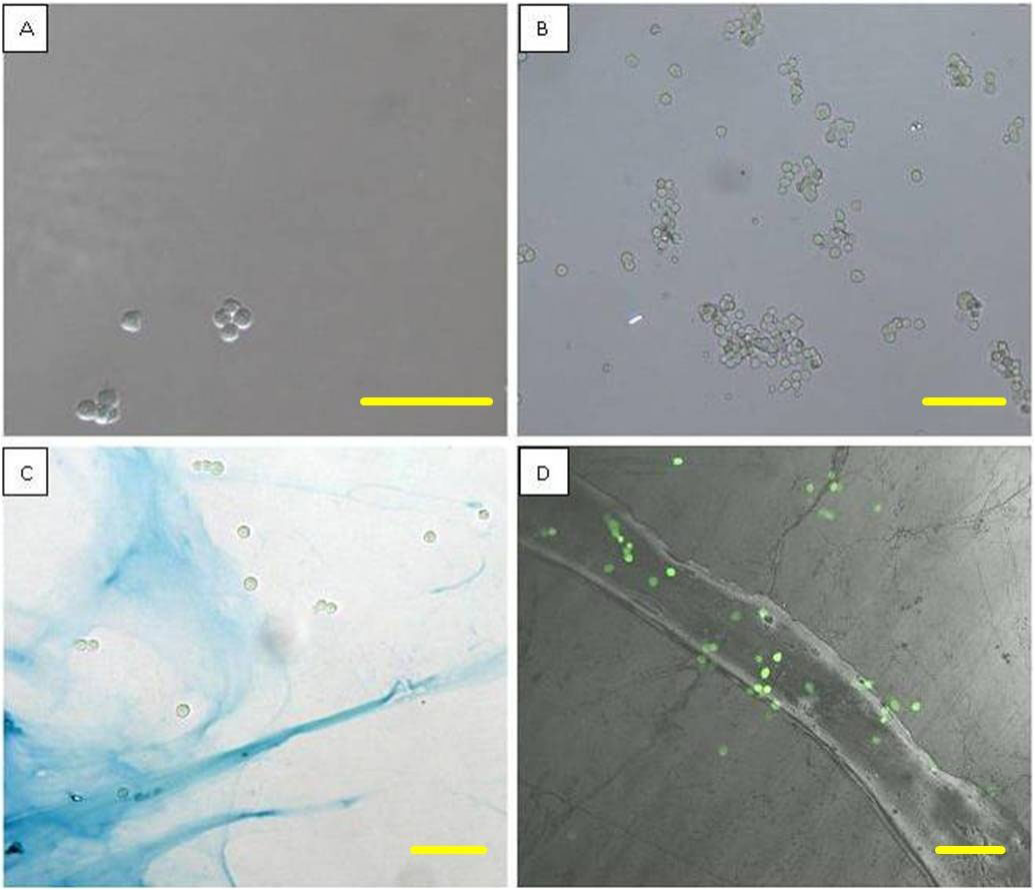
**Light and confocal micrographs of PC12 cells attached to various substrate surfaces**. A) Negative control sample with PC12 cells loosely attached to a bare glass surface. B) Positive poly-L-lysine coated glass surface, with numerous attached PC12 cells. C) Light micrograph showing PC12 cells attached to a glass coverslip coated with Sundew adhesive. The coverslip was stained with Alcian Blue to demonstrate the pattern of the coating, and the association of the cells with the scaffold. This micrograph shows a sparse area of attachment that allows clear delineation of individual cells, and a typical coating pattern. Areas with more complete coating had a greater number of attached cells. D) Confocal micrograph displaying a thick area of Sundew adhesive, and the thinner networks branching off from the thickly coated area. The PC12 cells were stained with calcein to determine viability, green equaling viable cells, and it is easy to observe the cells attaching to the Sundew adhesive coated areas. Scale bar = 25 μm.

All experimental studies consistently confirmed that the negative control had an average of 6 ± 1.3 cells attached per field of view, which was much less than the poly-L-lysine coated control that had 66 ± 4 cells attached per field of view. The Sundew adhesive coated sample had 49 ± 6 cells attached per field, significantly more than the untreated control (Figure 8). T-tests conducted on the data showed a significant difference between all samples with p values < 0.01. Calculating the number of attached cells per mm^2 ^yielded 147 cells/mm^2 ^for the negative control, 1681 cells/mm^2 ^for the positive control, and 1253 cells/mm^2 ^for the Sundew adhesive coated surface. Due to the non-uniformity in the coating of the Sundew samples, there was not as much surface area available for attachment compared to the positive control. This could lead to a bias in number of attached cells counted between these two samples. Little difference was observed, however, in the viability of the cells that attached in all samples. 92% of attached cells were viable in the negative control with 100% and 98% viable in the positive control and Sundew adhesive coated sample respectively. For all samples, the majority of cells displayed a round morphology and similar size. Without the addition of nerve growth factor, more cells appeared to take on a polar shape in the positive control and the Sundew adhesive coated surface, whereas no polar cells were observed in the negative control. As demonstrated in the Alcian Blue samples, PC12 cells attached to the Sundew adhesive coated surface and were most tightly associated with the stained scaffold (Figure 8C-D). The cells attached to the Sundew adhesive coated surface were subjected to vigorous rinsing to attempt to dislodge the cells, but the cells remained attached through this process indicating a stable attachment. The results from these experiments demonstrated the potential for the Sundew adhesive to be used for cell attachment in the field of tissue engineering. Based on the images obtained from these experiments, it appears that the PC12 cells favored areas with thinner coatings of the scaffold, as confirmed by both AFM imaging and the staining pattern of Alcian Blue coated surfaces. The cells generally attached to areas where the Alcian Blue staining was barely visible, which corresponded to thin layers (< 80 nm) of the nanonetwork. In the same manner, by being able to deposit a uniform pattern of coating, it may be possible to direct the growth of neurites into differentiated neurons. In essence, this study has demonstrated the potential for a novel material identified from nature to be used in the complex field of tissue engineering.

## Conclusions

Through this study, we have systematically examined the nanoscale structure of the adhesive generated from the Sundew, and evaluated the potential of this material to be used for tissue engineering. It was determined that the adhesive is a nanocomposite composed of water, nanoparticles, polysaccharide, and salts. This nanocomposite was observed in three species of Sundew, and was shown to form a network of nanofibers independent of the surface. When dried, this adhesive serves as a suitable substrate to promote the attachment of PC12 neuron-like cells, and may be used for a variety of other cell types. Further study into the role of the nanoparticles within the nanocomposite will lead to a better understanding of how nanoparticles can be used in adhesives. Experimentally, nanoparticles have been shown to help increase adhesion of epoxy adhesives [35]. The presence of nanoparticles in the Sundew adhesive may increase surface contact and generate larger force for initial binding to insects. Another possibility is that the nanoparticles may provide a mechanical support that allows the liquid polysaccharide to stretch beyond what has previously been observed. This could explain the high elasticity observed in the liquid adhesive. Moreover, the potential uses of composite materials from biological organisms show promises for a wide variety of applications [35]. A Sundew adhesive inspired biomaterial can be proposed for a wide range of biomedical applications. In addition to tissue engineering, it may be used for biological treatment of wounds, regenerative medicine, or helping enhance synthetic adhesives. Further studies will focus on extending the results obtained from this study to evaluate the additional potential for this material to be used in biomedical applications.

## Competing interests

The authors declare that they have no competing interests.

## Authors' contributions

MZ, SCL, LX, DL and WH designed the overall project. MZ, SCL and LX wrote the manuscript. WH, SCL, and DL helped with the interpretation of data and revised the manuscript. LX took care of the sample preparations and characterization of nanostructure of Sundew adhesive using AFM. WH characterized the elasticity of Sundew adhesive and contributed to the nanostructure characterization of Sundew adhesive. SCL and WH performed the PC12 study on the scaffolds. DL and XF contributed to the TEM characterization of Sundew adhesive. All authors read and approved the manuscript.
